# Shared mechanisms underlie mental imagery and motor planning

**DOI:** 10.1038/s41598-022-06800-9

**Published:** 2022-02-22

**Authors:** Rotem Bennet, Miriam Reiner

**Affiliations:** Technion, Israel Institute of Technology, Haifa, 3200003 Israel

**Keywords:** Sensorimotor processing, Cognitive control, Motor control

## Abstract

Many studies have associated mental imagery with motor control mechanisms by showing mutually active brain areas and functions, as well as similar temporal patterns of imagining and executing the same motor actions. One of the main conjectured mutual mechanisms is the Cerebellar forward-model, commonly believed to generate sensory predictions as part of both motor control and mental imagery processes. Nevertheless, trials to associate one’s overall individual mental and motor capacities have shown only mild and inconsistent correlations, hence challenging the mutual mechanism assumption. We hypothesized that one cause to this inconsistency is the forward-model’s dominance in the motor-planning stage only when adapting to novel sensorimotor environments, while the inverse-model is gradually taking the lead along the adaptation, and therefore biasing most attempts to measure motor-mental overlapping functions and correlate these measurements under regular circumstances. Our current study aimed to tackle and explore this gap using immersive virtual embodiment, by applying an experience of a fundamental sensorimotor conflict, thereby manipulating the sensory prediction mechanism, and presumably forcing an increased involvement of the forward-model in the motor planning stage throughout the experiment. In the study, two groups of subjects (n = 48) performed mental and manual rotation within an immersive, motion-captured, virtual reality environment, while the sensorimotor dynamics of only the test group were altered by physical-virtual speed re-mapping making the virtual hand move twice as fast as the physical hand controlling it. Individual mental imagery capacities were assessed before and after three blocks of manual-rotation, where motor planning durations were measured as the time until motion onset. The results show that virtual sensorimotor alteration extremely increases the correlation of mental imagery and motor planning (r = 0.9, p < .0001) and leads to higher mental imagery performance improvement following the physical blocks. We particularly show that virtual embodiment manipulation affects the motor planning stage to change and functionally overlap with imagery mechanisms, rather than the other way around, which supports our conjecture of an increased sensory-prediction forward-model involvement. Our results shed new light on the embodied nature of mental imagery, support the view of the predictive forward-model as a key mechanism mutually underlying motor control and imagery, and suggest virtual sensorimotor alteration as a novel methodology to increase physical-mental convergence. These findings also suggest the applicability of using existing motion-tracked virtual environments for continuous cognitive evaluation and treatment, through kinematic analysis of ongoing natural motor behaviors.

## Introduction

Evidence has accumulated in the last few decades, showing significant similarities between various quantitative parameters of physical actions and mental imagination of performing the same actions^1–4^, similar involuntary physiological responses^5–7^, as well as mutually facilitating or interfering interactions between temporally proximal motor and mental activities^8–13^. Brain imaging studies also show that during mental imagery there is brain activity typical to what can be seen during the various stages of motor preparation and execution, and joint mechanisms which are typically part of motor control loops become active^14^. Imaging studies have also revealed separate representations of feedback vs. feedforward control, with mental imagery activity overlapping substantially with open-loop control activity, and separate from closed-loop control activity which is associated more with feedback control areas^15,16^. These serve as a strong support for the currently held belief about the mutual brain mechanisms underlying both motor control and mental imagery processes. Nevertheless, many of these findings may be explained as indicating similarity of neural computation principles (e.g. sequential and analog simulation), as well as reliance on joint underlying basic functions (e.g. neural oscillators) and brain areas, and not necessarily stem from actually sharing mutual brain modules which serve both mental and motor processes. This dissociates claiming for mental-motor functional and computational “equivalence”^17^ from the stronger claim of mental-motor functional “sharing” of brain circuits which are at the core of both mental imagery and motor control mechanisms. Tackling this potential gap is one of the goals of our current research, which uses inter-subject co-variance of mental and motor individual capacities, as an assessment of truly mental-motor “sharing” of core underlying brain mechanisms.

Most of the mental-motor similarities and interactions found so far, were using a task-wise, within-subject, analysis, where respective mental and motor processes are correlated across the tasks of each subject^3,4,18,19^. Critically, this puts more weight on explaining the between-task rather than between-subject variability using the co-varying mental-motor measurements. Statistically, this means that all the task-specific parameters should be regarded as “random factors” and are otherwise potentially confounding the interpretation of any found mental-motor connection as reflecting a joint underlying mechanism mutually serving mental-imagery and motor-control. One other way to address this potential task-specific confounding factors, is to calculate a grand-average of performance metrics across all the tasks or task-parameters, as overall individual capacity assessments, and analyze the covariance (or correlation) between capacities of interest across all the experiment participants. Many studies have used this psychometric approach^20–22^, and this was also the methodology used in our own study, as the average motor-planning durations are correlated with the average mental-imagery RT’s. Such correlations, and the calculated percentage of between-subjects variance they explain (the R-squared), strongly indicate the extent of overlap, or sharing, between the brain mechanisms underlying the correlated individual capacities.

Studies comparing the individual capacities of mental-imagery and motor-control, as in our research, report varying degrees of correlations, mostly depending on the task and measurement used in the experiment. In a study that compared individual speeds of mental-rotation and visuo-motor rotation, using a motor task somewhat comparable to the one used for our own control-group (moving a computer mouse to match a presented angle), the correlation found between the capacities was in the range of r = 0.65–0.69^21^. Another study found correlation of r = 0.38 between assessed motor coordination-skills and mental- rotation capacity of adults^23^. When comparing several motor-development and mental-rotation capacities of 5–6 years old children, the correlations ranged between 0.16–0.44, with the motor items of balance (r = 0.39), body-agility (r = 0.37) and motor-control (r = 0.36) correlating the most with mental-rotation^24^. Higher mental-motor correlations, of up to r = 0.59, were found in a follow-up study with 3–4-year-old children^25^. While most of these studies assessed the individual motor capacity through measuring the performance of the actual movements, a recent paper has suggested using the motor-planning stage specifically as the most relevant motor performance metric to correlate with mental imagery, and found correlation of r = 0.42 between motor planning efficiency (accuracy) and motor imagery capacities for 6 year old children^26^.

Sensorimotor prediction mechanisms are essential components of the motor system control loop, allowing the generation of smooth, fast and error-minimizing motor actions^27–29^. These mechanisms have been increasingly studied, most notably within the predictive-coding framework^30^. It has been proposed that they underlie both motor control and cognitive processes. Predictions are, presumably, calculated by the cerebellar forward-model, described as a form of a sensory simulator, which, given an intended motor command and the existing sensory-motor context, generates a prediction of the expected sensory outcome. Such model is presumed to be implemented as part of both the motor-planning stage, throughout the actual motion execution, and during mental imagery^29,31^. A complementing model, termed the cerebellar inverse model, has been proposed as well. This model receives the intended trajectory as its input and then rapidly generates the motor command, required to achieve it, which, in turn, is transferred to the muscles. Biological motor control research describes the motor system as composed of multiple pairs of inverse and forward internal models^27^, with the inverse-model being more dominant in fully trained motion, familiar contexts and automatic movements, and the forward-model being more active in stages of motor adjustment to new sensorimotor contexts^32^, acting as a training-signal generator for the inverse-model’s longer and slower learning and adaptation. Based on these theories we reasoned that, under conditions of novel or rapidly changing environment, where sensorimotor adaptation is required, an increased involvement of the forward-model mechanism in motor planning will be induced.

**Figure 1:**
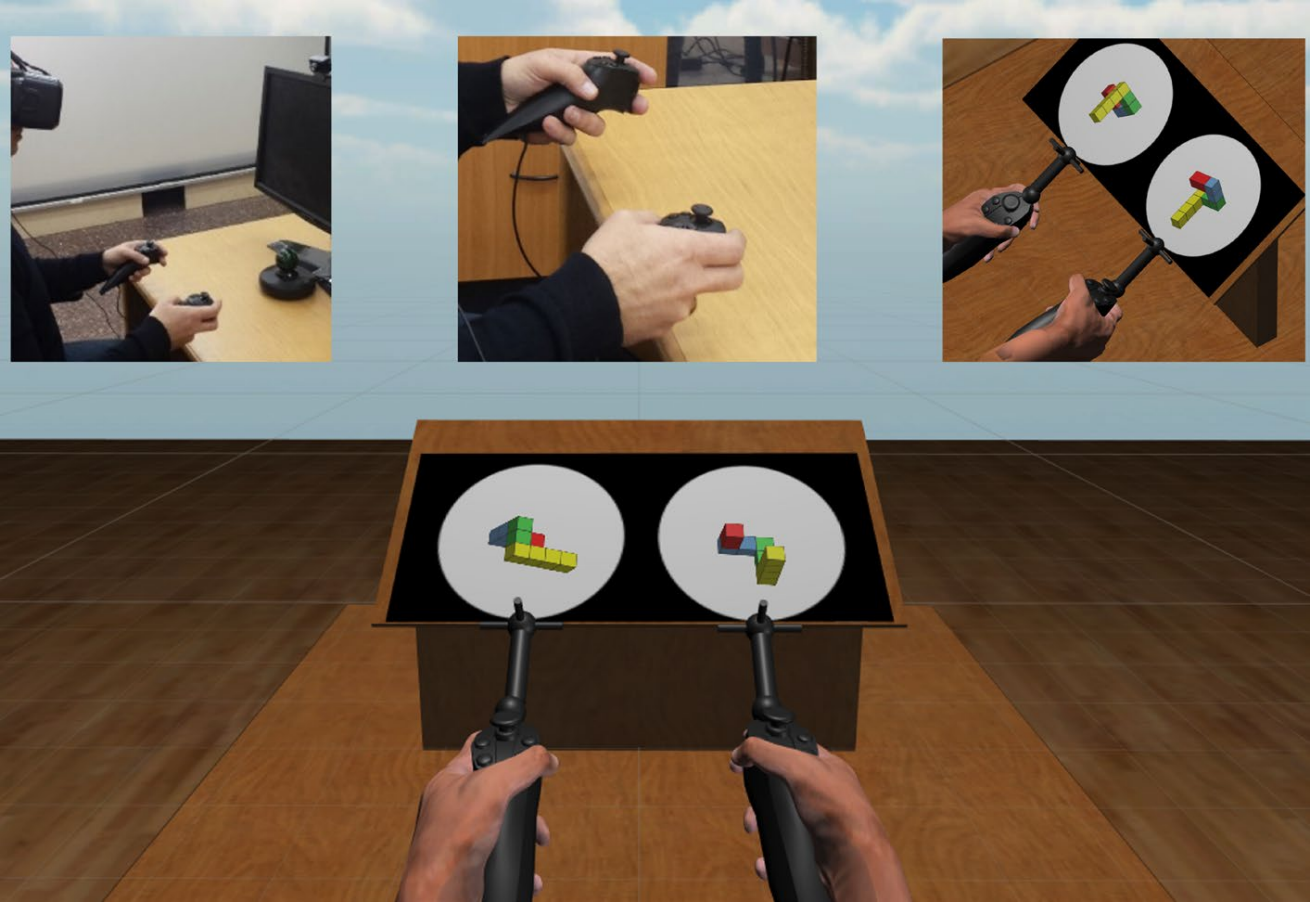
The virtual-reality scene from the participant’s point of view (bottom), and the experimental setup consisting of a virtual-reality headset, earphones and two handheld motion-tracking controllers (top-left), with the physical hands holding the physical controllers (top-middle) always experienced as posturally matching the virtual hands holding the virtual controllers (top-right).

The main motivation to the current study was that it is now widely accepted that mental imagery relies to a large extent on the sensory prediction mechanism highly similar in principle to the forward-model. Furthermore, imagery can be viewed as the internal cycles of the top-down copies of the motor commands and their resulting sensory experiences, predicted by the forward-model, while inhibiting the actual motor execution. We thus hypothesized that, in addition to inducing larger involvement of the forward-model in motor planning, novel or rapidly changing environment will also result in an increased overlap with the same mechanisms used for mental imagery. To test this hypothesis we created a novel sensorimotor environment, in which an initiation of sensorimotor adaptation could be induced. The setup consisted of an immersive virtual reality headset, earphones and two handheld motion-tracking controllers. The participants wearing the headset experienced as if they are sitting in front of a table, holding two controllers similar to the physical ones with realistic renderings of virtual hands constantly tracking their physical motions, thus keeping their virtual and physical hands posturally congruent along the experiment, and getting realistic tactile feedback from the virtual controllers due to holding exactly the same controllers in reality as well. By this we assumed the introduction of an immersive embodiment, identifying the virtual hands as one’s own hands (ownership) and as controlled by one’s own motion (agency), thereby imposing a virtual sensory outcome, of the virtual hands always in view, into the motor control loop, instead of the expected sensory outcome of the physical hands hidden from view. Following the wealth of previous literature on the necessary and sufficient conditions for virtual embodiment^33–38,38–42^, and as the hands’ physical-virtual posture, motion, control and tactile feedback were all perfectly synchronized in our setup, we therefore chose not to explicitly measure the level of “embodiment”, and assume the effectiveness of the setup for the purpose of our study (which also got supported, after the fact, by subjective spontaneous responses from the participants). The controllers within the virtual reality scene were connected as handles to a setup where the right handle’s rotations could rotate the image of a 3D shape, projected on a 2D display, as if the shape is connected to the handle. See Fig. 1.

**Figure 2:**
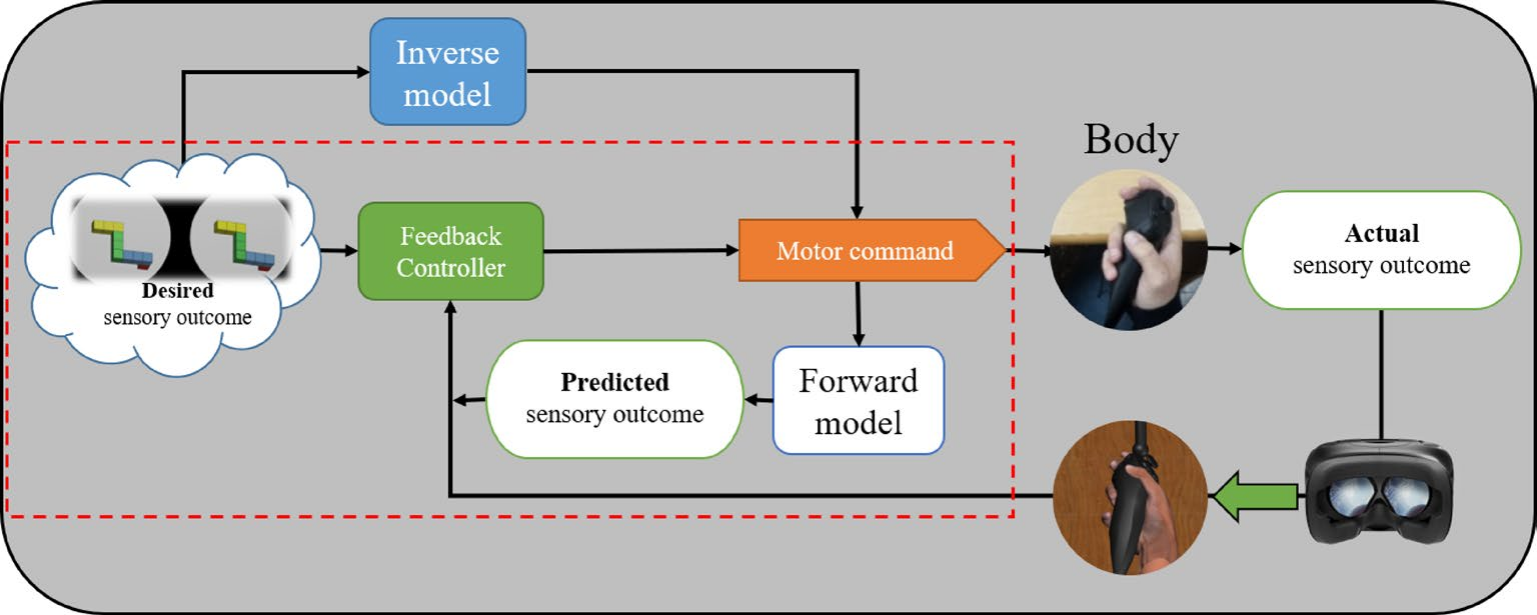
Motor control-loop model of the experiment, following the current view of the neural motor-control feedback loop combining Forward and Inverse models. The red rectangle marks the assumed planning/imagery part.

As the experimental manipulation, we altered the predicted motion dynamics by changing the virtual hand’s speed. The scheme of the motor control-loop, assumed to be implemented in our experiment for motor planning, operation and imagery, is illustrated in Fig. 2. We hypothesized that under the conditions of sensory (virtual hand) and motor (unexpected speed) novelty, the impact of the forward-model on constructing the motor plan would be much more dominant than that of the inverse-model, leading to an increased similarity between the individual motor planning and mental imagery. We further assumed that this increased mutual reliance on the forward-model will be manifested in speed similarities between the motor planning and the mental imagery. To evaluate the extent of speed similarity, we tested two groups of subjects, one submitted to the altered sensorimotor environment, and one for which there was no physical-virtual remapping, presumably allowing the utilization of the existing inverse-model for motor planning.

**Figure 3:**
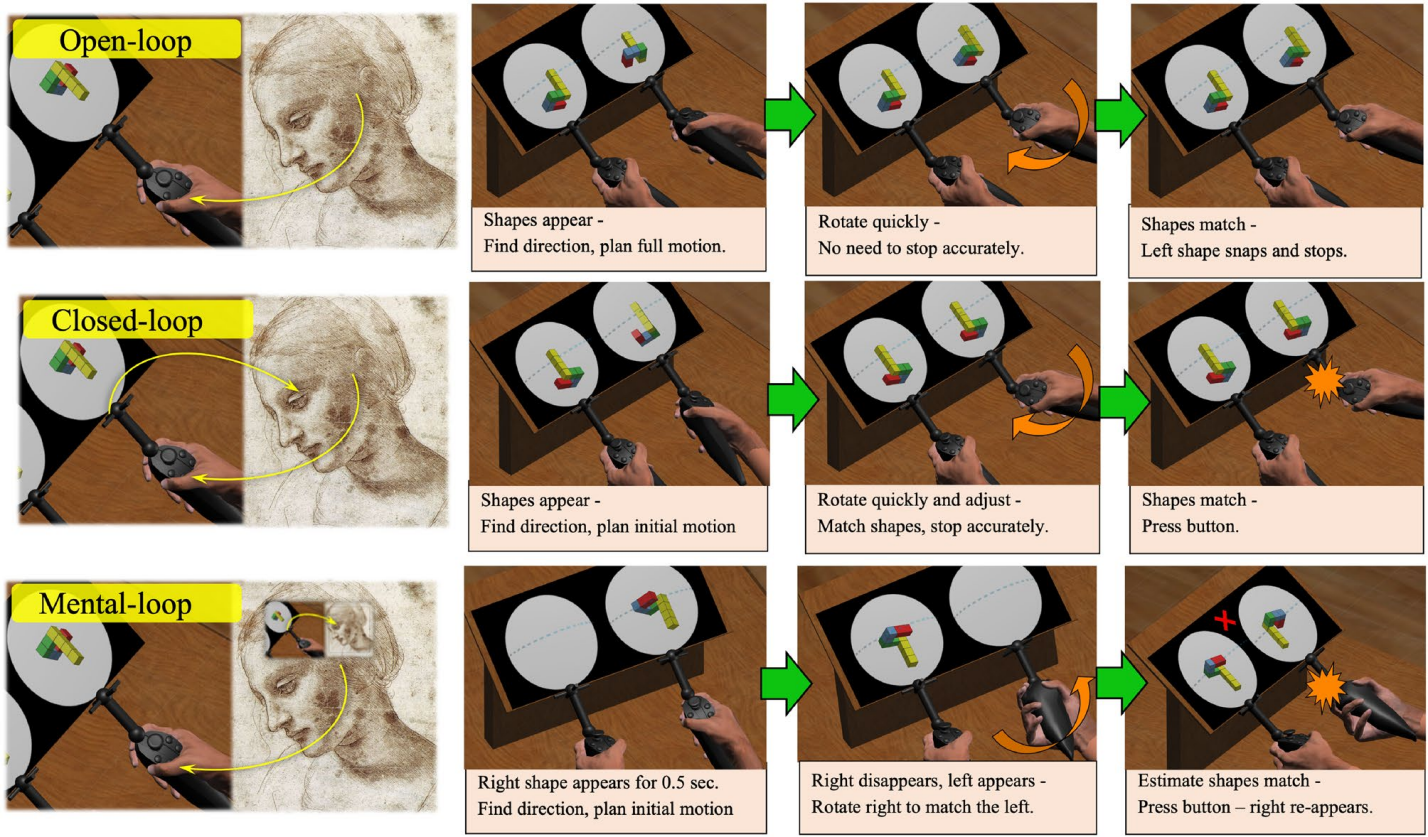
Trial structure in each of the motor-blocks: open-loop (top), closed-loop (middle) and mental-loop (bottom).

The experiment consisted of three types of motor blocks, which shared the participant's goal of rotating the right shape until it matched the left shape’s rotation, but differed in the required motor control task, as illustrated in Fig. 3. In one type of block, termed “open-loop”, no online control was required, as the right shape automatically stopped its motion when reaching the left shape’s rotation, thus requiring the subject to just decide on the rotation direction and initiate a fast movement until the shape “snaps” to its place, which was presumed to put more weight on fully completing the motor plan before the motion onset. Another, termed “closed-Loop” required online control of the rotating virtual hand, in order to stop the rotation exactly when the right shape reaches the left shape’s rotation. The third, “mental-loop”, required online control, yet without visual feedback of the right shape’s ongoing rotation, thereby requiring an online mental imagery during the rotation itself. These conditions were designed to maximize the accuracy of the individual motor planning speed measurements, and to reduce the possible inter-subject variability of relying on online feedback during the actual motion. Relying on online feedback may lead to initiation of the actual motion before the motor plan is fully completed, thus impeding the evaluation of the individual speed of the required motor planning. We hypothesized that open-loop control would provide the most accurate measure of the motor-planning, since in the effective absence of visual feedback as relevant in the motor-control loop, the motor-plan should be constructed to its fullest before motion-onset. In the closed-loop blocks, we assumed that the motor-plan is only partly completed prior to movement execution, given that it gets refined throughout the motion itself and thus need not be fully planned in advance.

**Figure 4:**
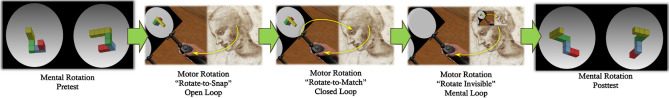
The experiment’s trial sequence, from left to right, beginning with mental-rotation pretest, followed by the three motor-control blocks, and ending with another mental-rotation posttest.

The motor blocks were preceded and followed by pretest and posttest mental-rotation blocks, respectively. In these blocks, 2 randomly rotated stimuli (same or mirror) were presented side by side on each trial. Participants were asked to decide as quickly as possible whether each pair consisted of the same stimuli or two mirrored ones. The experiment’s trial sequence is illustrated in Fig. 4.

This study aimed to test, and possibly demonstrate, that under sensorimotor adaptation conditions, which induce an increased reliance on a forward-model, similarity between motor planning and mental imagery will be more pronounced. In particular, we aimed to answer the following research questions:

1. Do mental-imagery and motor-planning mechanisms overlap and can we measure the extent of it?
2. Do mental-imagery and motor-planning mechanisms overlap more under sensorimotor adaptation?
3. Do mental-imagery and motor-planning mechanisms overlap more in open-loop motor tasks?

To provide evidence that, to some extent, motor-planning and mental-imagery operate on a shared mechanism, beyond being functionally or computationally “equivalent”^17^, we set out to test the inter-subject correlations between the individual motor-planning and mental-imagery capacities, as reflected by their average speed across all the trials. Such inter-subject correlation would indicate the extent to which the mental imagery and motor planning co-vary between the subjects. The magnitude of this between-subject co-varying, namely, the coefficient-of-determination (the R-squared) is indicative of the extent of mechanism sharing.

The experiment was administered to two groups of subjects, differing by the speed of the virtual hand operated by them in the virtual reality scene. The control group performed the tasks with a virtual hand tracking the physical hand’s rotation speed precisely, thereby without any sensorimotor adaptation needed (no physical-virtual mismatch), whereas the test group performed the tasks with the virtual hand programmed to be twice as fast as the physical hand, imposing novel sensorimotor context which thus requires adaptation. The mental-motor individual-capacity correlations were analyzed for each of the three motor-control blocks (open-loop, closed-loop, mental-loop), and were compared between the groups.

## Results

### Mental-Rotation RT: Mental simulation, Individual imagery speed and Inter-subject variability

Subjects’ RTs obtained under the pretest and the posttest mental-rotation blocks were measured. RTs of the correct trials were submitted to a mixed-effects analysis with group (altered hand speed, regular hand speed) as a between-subjects factor, and shape’s continuous angular-disparity angle (angular-disparity: 10, 60, 110, 160) as a within-subject factor. Pretest and posttest mental-rotation RTs were analysed separately. In the pretest mental-rotation block, the fitted full model explained 63.7% of the overall RT variance (adjusted R-squared), indicating that the included factors could partially explain the RTs’ distribution. The RTs linearly increased with an increase in the angular disparity, and followed the Shepard and Metzler’s (1971) Angular-Disparity Effect (ADE). This linear dependence (F(1, 15.87) = 113.37, p< .0001) suggests that participants indeed imagined an actual rotation. There was no main effect of the group (F(1, 46.04) = 0.23, p = .63), as well as no group x angular-disparity interaction F(1, 47.34) = 3.70, p = .06), indicating that the RTs as well as their dependence on the angular disparity were not sensitive to the artificial hand speed manipulation. Similar effects remained in the later posttest block. The full model explained 60.9% of the overall RT variance (adjusted R-squared), with a significant main effect of angular-disparity on RT (F(1, 17.98) = 74.78, p< 0.0001). No main effect of group, F(1, 45.91) = 0.13, p = .27, nor interaction of it with angular disparity (F(1, 42.56) = 0.20, p = .66) were found. These results show that the test blocks had no impact on the insensitivity of RTs to the artificial hand speed manipulation, nor did they induce an effect of artificial hand speed manipulation on RTs’ dependence on the angular disparity.

We have assumed that our participants should vary in their mental-rotation capacity, impacting the ADE slopes and intercepts. We have accounted for this inter-subject variability in the above fitted mixed-effects models. In the pretest block, the estimated mental-rotation capacity variation between individuals explained as much as 34.18% of the total variance. Similar results were obtained in the posttest block, where the inter-subject capacity variation accounted for 33.1% of the overall measured RT variation. These results indicate that about half of the explanatory power of the model stems from inter-subject variability in their mental-rotation capacity. Importantly, this reflects the population mental-rotation capacity variation across the experimental groups. To further test the extent and statistical significance of the inter-subject mental-rotation capacity variability, we submitted the mental-rotation blocks RT’s to an ANOVA analysis, with the subjects and the angular-disparity as independent variables. The model fitted for the pretest block explained 48% (R-squared adjusted) of the overall variance, with a significant effect of the Angular Disparity (F(1, 812) = 340.06, p< 0.0001), indicating the cross-subjects sensitivity to changes in the required angle of movement, and a significant effect of the subjects (individual ADE intercepts, F(47, 812) = 9.25, p< .0001), indicating that this sensitivity is characterized by individual rather than general dynamics. A significant interaction between subjects and angular-disparity (subjects individual ADE slopes, F(47, 812) = 2.49, p< .0001), suggests that the relationship between the subjects and their sensitivity to changes in the required angle of movement is not random but rather regular. Similar effects were found for the posttest mental-rotation RTs as well. To compare the inter-subject variability between the pretest and the posttest blocks, the mean RTs across all angles in the pretest (M=3.34s, SD=1.09) and the posttest (M=2.40s, SD=0.75s) blocks were calculated for each subject, and the correlation between the pretest and the posttest RTs was calculated. A high correlation (r=0.88) was demonstrated between the pretest and the posttest RTS, reflecting high test-retest reliability of about 77%.

### Motor-planning duration predicts later actual motion kinematics

To test the presumed relation between the duration of the motor planning period and the following executed motion in each of the motor-control blocks, we submitted the actual shape rotation angle to a mixed-effects analysis, with the subjects as a random factor, hand speed manipulation (altered, regular) as a between-subjects factor and before-motion duration as a within-subject continuous variable. In the closed-loop block no significant main effect of hand speed manipulation (F(1, 45.6) = 2.39, p = .13) nor significant main effect of before-motion duration (F(1, 1621) = 1.78, p = .18) were found. Their interaction was not significant as well (F(1, 1621) = 1.62, p = .20). However, applying ANOVA to the mental-loop block’s rotation angles revealed a significant main effect of the hand speed manipulation (F(1, 44.7) = 4.81, p = .034), indicating that the mean rotations were significantly larger in the test group (M = 63.78 degrees, SE = 2.04 degrees) than in the control group (M = 57.46 degrees, SE = 2.04 degrees). The main effect of the before-motion duration yielded F ratio of F(1, 1551) = 8.63, p = .003, indicating a significant positive linear relation between the motor-planning durations and the following rotation motion. The interaction effect was not significant (F(1, 1551) = 0.31, p = .58), meaning that the discovered linear relation between the planning durations and the actual motion was not significantly different between the two groups. Similarly, ANOVA on the open-loop block revealed a main effect of the hand speed manipulation (F(1, 45.4) = 14.93, p = .0004), indicating that the mean rotations were significantly larger in the test group (M = 80.46 degrees, SE = 2.05 degrees) than in the control group (M = 69.26 degrees, SE = 2.05 degrees). The main effect of the before-motion duration yielded F ratio of F(1, 1736) = 14.78, p = .0001, indicating a highly significant positive linear relation between the motor-planning durations and the following rotation motion. The interaction effect was not significant (F(1, 1736) = 0.61, p = .43), meaning that the linear relation between the planning durations and the actual motion was not significantly different between the two experiment groups in this type of block as well.

To further assess the relation between the duration of the motor planning and the actual movement, the actual rotation durations rather than the rotation size (angle) were submitted to a similar analysis. Applying ANOVA to the closed-loop block durations revealed a significant main effect of the hand speed manipulation (F(1, 45.3) = 5.14, p = .028), indicating different rotation-durations for the test group with the altered hand speed (M = 461.47ms, SE = 33.04ms) as compared to the control group (M = 567.34ms, SE = 33.03ms). This result was expected given that the speed of motion of the artificial hand was increased in the test group. The main effect of the before-motion duration was not significant (F(1, 1865) = 0.04, p = .836), as well as the interaction between the two factors (F(1, 1865) = 0.23, p = .634), indicating that the closed-loop before-motion planning duration had no impact on the duration of the later motion, without significant difference between the speed manipulation groups. In the mental-loop block, the main effect of the hand speed manipulation was non-significant (F(1, 45.7) = 0.24, p = .629), whereas the main effect of the before-motion duration was significant, (F(1, 1819) = 21.41, p< .0001), indicating a positive linear relation between the planning duration and the eventual rotation duration. The interaction effect was not significant (F(1, 1819) = 0.19, p = .664). In the open-loop block, the main effect of the hand speed manipulation was significant (F(1, 45.8) = 13.17, p = .0007), reflecting significantly different rotation durations between the groups (test group: M = 145.94ms, SE = 11.91ms vs control group: M = 207.09ms, SE = 11.91ms). In this block as well, the main effect of the before-motion duration was significant (F(1, 1893) = 113.02, p< .0001), but the interaction was not (F(1, 1893) = 0.36, p = .547).

### Individual motor-planning and mental-rotation capacities are strongly correlated

**Figure 5:**
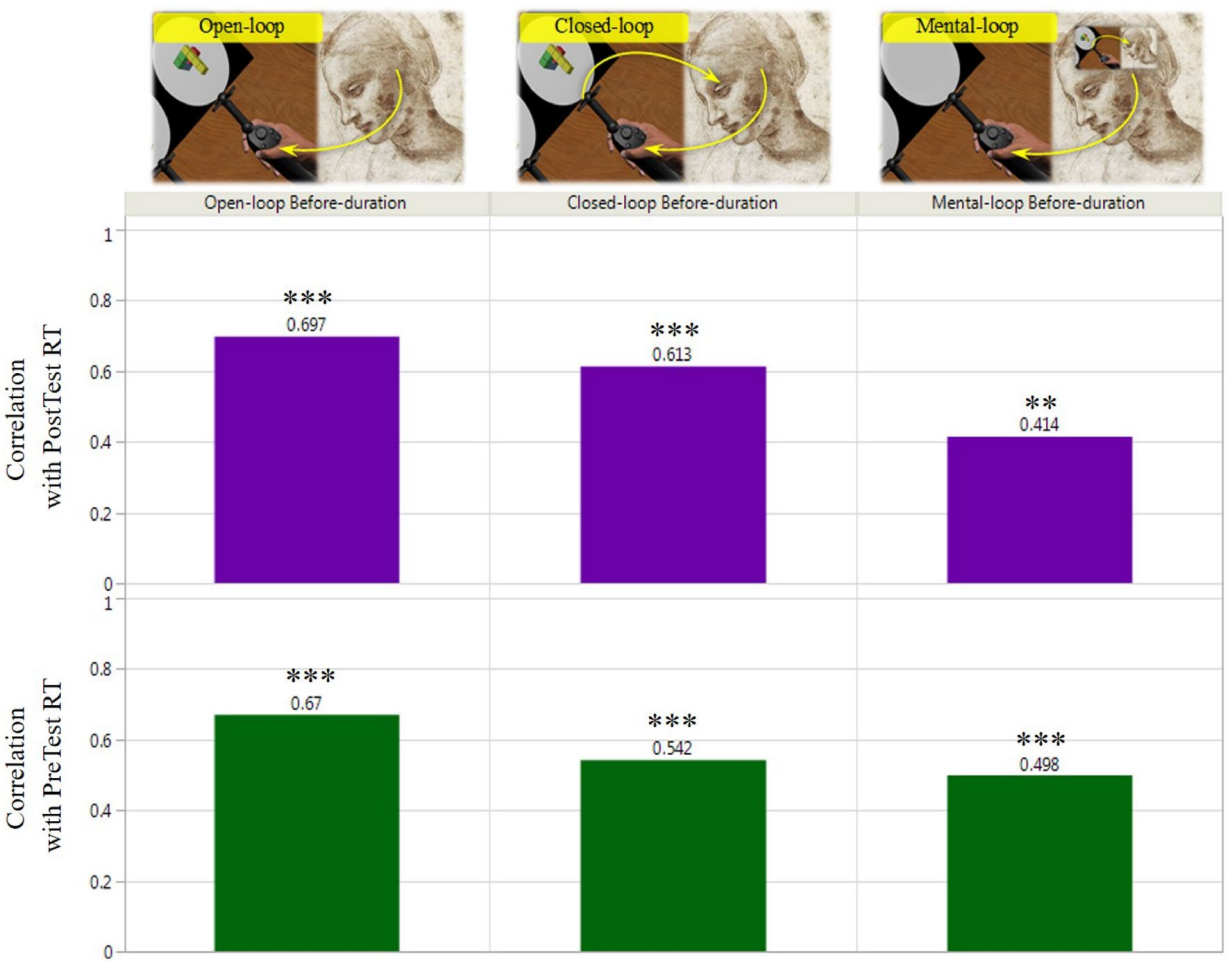
Inter-subject mental-motor capacity correlations. Pearson’s r correlations between individual average motor-planning and mental-rotation durations, per motor-control mode and mental-rotation pretest/posttest blocks (top). Statistical significance levels are denoted with Asterisks.

We hypothesized that under the conditions that impose novel sensorimotor context, the existing overlap between the mechanisms underlying motor planning and imagery will be augmented. We expected that 1. This overlap will be reflected in correlations between individual mental and physical capacities. 2. The correlations will be further augmented under the sensorimotor alteration conditions. To test this hypothesis, we calculated individual average before-motion duration times (motor-planning capacity) for each of the motor-control blocks, and individual average mental-rotation RTs for the pretest and posttest mental-rotation blocks (mental-imagery capacity), relying on the common assumption that these average durations indeed represent assessments of motor-planning and mental-imagery capacities, respectively^19,43–45^. We then calculated the correlations between the individual motor and imagery assessments for each motor-control block and tested their statistical significance. The overall results are presented in Fig. 5. As can be seen in the graph, strong correlations were found between the individual capacities (average speeds) of mental-imagery and motor-planning, with the highest correlations of r = 0.67–0.7 measured in the open-loop motor block. To our knowledge, our findings demonstrate higher correlations than typically found in other studies comparing mental-imagery and motor-planning capacities^26^. Our correlations are comparable to the high-end of the reported correlation range (r = 0.36 - 0.69) found in studies measuring mental-imagery and general motor capacity, rather than specifically motor-planning^21,46^.

### Sensorimotor alteration increases similarity of mental-motor capacities

**Figure 6:**
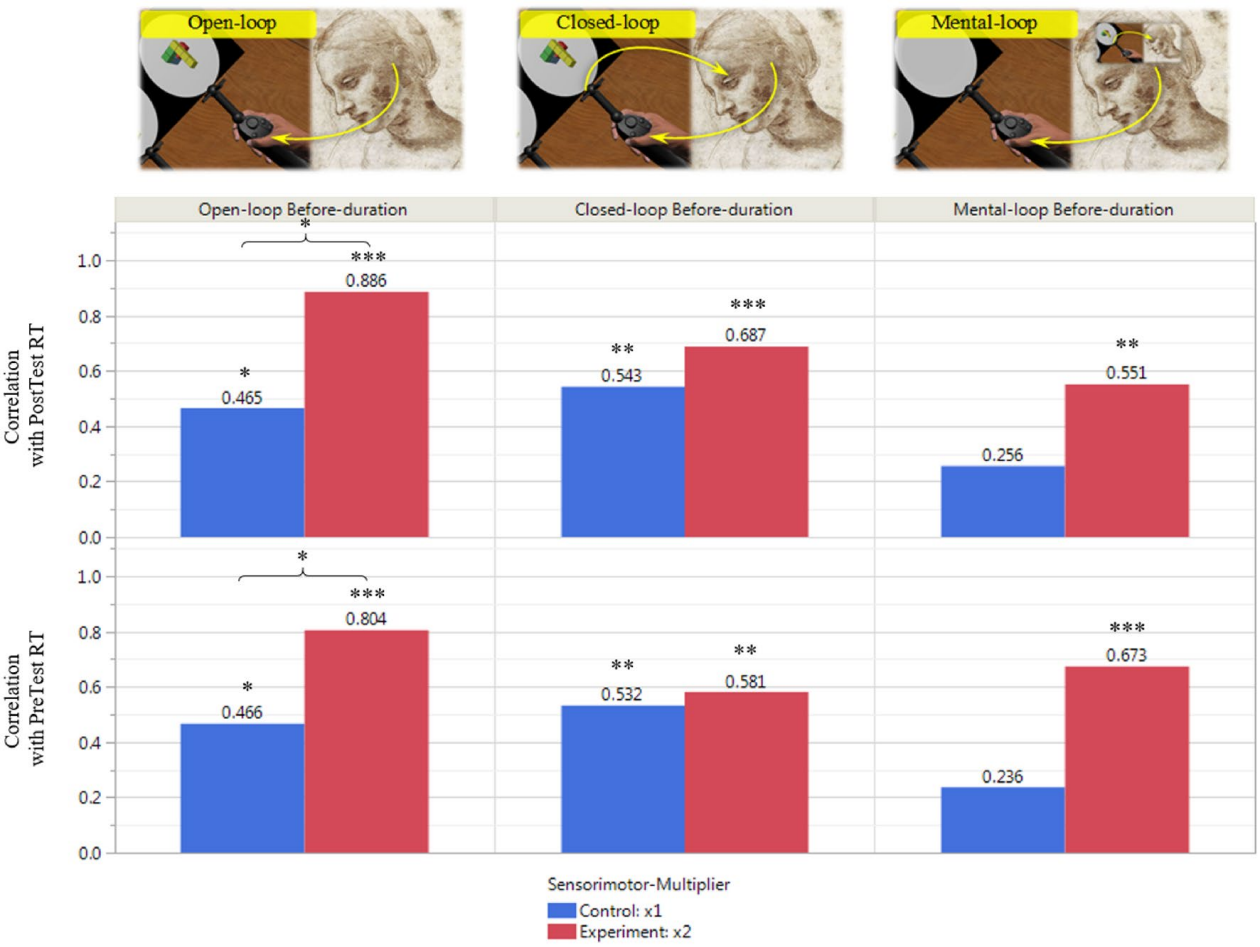
Correlations of mental-motor individual capacities by condition group. Pearson’s r correlations between individual motor-planning and mental-rotation mean durations, per motor-control mode and mental-rotation pretest/posttest blocks, separately for each Sensorimotor-Multiplier (virtual speed alteration) condition group of subjects. Statistical significance levels are denoted with Asterisks.

To demonstrate that introducing novel sensorimotor context (the hand speed manipulation) augmented the overlap between the motor planning and imagery, the same capacity correlations were calculated, separately for each study group. The correlations are shown in Fig. 6, with statistical significance marked with Asterisks. As can be seen in the correlations graph, very strong correlations of up to r = 0.89 were found for the test group subjects between their individual open-loop motor-planning and mental-imagery capacities, both when correlated with the pretest as well as the posttest mental-rotation blocks. These high correlations suggest that, under these virtual settings, measuring this single motor parameter provides a close estimation of the individual mental-imagery capacity, as  81% of the inter-subject mental-imagery variability is explained by the individual variability in the measurement of this open-loop motor performance. Though not statistically significant, a general trend of higher correlations in the test group as compared to the control group was found in the other types of blocks as well, suggesting that some aspects of the study manipulation affect motor-planning in general. Importantly, the similar correlation of the test group mental-motor capacities was found with both the posttest mental capacity, after the motor blocks, and the pretest mental capacity, where no sensorimotor manipulation has yet been applied. This implies that the manipulation has affected the motor-planning mechanisms to overlap and become temporally more similar to the mental-imagery mechanisms, rather than vice versa. This is in line with our hypothesis that sensorimotor alteration increases the need in finer sensory prediction at the planning stage, particularly in open-loop control, thus leading to increased similarity and overlap with motor-imagery processes, which inherently rely on presumably the same sensory prediction neural mechanisms (the cerebellar forward-model).

### Post-hoc: Motor training with sensorimotor alteration enhances individual imagery capacities

In addition to the main research question regarding the principal physical-mental inter-relation and the effect of the experimental sensorimotor manipulation on it, we were interested whether the manipulation in conjunction with the experimental structure allows for learning to take place, which might result in an improvement of individual mental-imagery capacity between the pretest and the posttest mental-rotation RTs. To this end, we calculated for each subject the difference between the individual mean mental-rotation RTs in the pretest and posttest blocks (mean-pretest minus mean-posttest), and submitted these differences to a one-way ANOVA with the hand speed manipulation (altered, regular) as the independent variable. The results showed that there was no significant difference between the experimental groups in their RT improvement between the pretest and posttest mental-rotation blocks (test group, M = 992.5ms, SE = 115.8ms; control group, M = 882.3ms, SE = 115.8ms; F(1, 46) = 0.45, p = .504). However, due to the previously observed high inter-subject variability in RT results, we suspected that the individual pretest-posttest RT differences might be determined by the initial pretest mental-rotation capacity (a floor-effect). To test whether pretest mental-rotation capacity consistently impacts the pretest-posttest RT differences, we fitted the individual pretest-posttest RT differences with the pretest individual mean RTs and the hand speed manipulation. We found that the fitted model explains ~60% of the inter-subject pretest-posttest RT differences variability (R-squared = 0.601), with a highly significant main effect of the pretest initial mental-rotation capacity on the subsequent improvement (F(1, 44) = 52.4, p< .0001), indicating that the pretest capacity indeed dictates the potential improvement following re-test. No significant main effect of the hand speed manipulation (F(1, 44) = 0.31, p = .578), and no significant interaction (F(1, 44) = 0.51, p = .481) were found, indicating that the improvement in RT was not impacted by the sensorimotor manipulation, yielding similar results for the test and the control groups.

To account for this conjectured individual floor-effect, we then calculated the 3-quantile values of the subjects’ pretest-RTs for each group and divided each group into 3 sub-groups of subjects according to their individual pretest-RT. Re-fitting the pretest-posttest RT differences with the hand speed manipulation (altered, regular) and the 3-quantile partitioning, revealed that the three sub-groups produced different patterns of improvement (main effect of the 3-quantile partitioning, F(2, 42) = 17.85, p< .0001). There was no main effect of hand speed manipulation (F(1, 42) = 0.84, p = .364), yet a significant interaction between the experimental group and the 3-quantile partitioning was found (F(2, 42) = 3.81, p = .030), indicating that the difference in the pattern of improvement between the 3 quantiles depended on the experimental group. Post-hoc Tukey’s HSD analysis of the 3-quantile sub-groups showed that the RT improvement of the subjects with the slowest pretest-RTs (the upper quantile, M = 1425.3 ms, SE = 104.1 ms) was significantly higher than the middle (M = 816.0 ms, SE = 104.1 ms) and the lower (M = 571.0 ms, SE = 104.1 ms) quntile sub-groups, which were not significantly different from each other. Post-hoc analysis of the interaction effect revealed that the pretest-posttest RT differences of the slower sub-group (upper quantile, M = 1712.9 ms, all the groups SE = 147.2 ms) was significantly higher than both the middle (M = 726.5 ms) and the lower (M = 538.2 ms) quantile sub-groups within the test group, as well as significantly higher than the pretest-posttest RT differences of the middle (M = 905.4 ms) and lower (M = 603.8 ms) quantile sub-groups of the control group. Participants from the upper quantile of the control group (M = 1137.6 ms) were not significantly different from the other qunatile sub-groups of the control group, and did not have lower pretest-posttest RT differences as compared to the upper quantile subjects of the test group.

Following these results, which validate the cognitive improvement floor-effect, we went on to narrow the RT-improvement comparison between the speed-alteration groups to only the upper 3-qunatile subjects of both condition groups, who are those with the initially highest pretest mental-rotation mean RT (i.e. those who have the most room for a cognitive improvement). Such a separate analysis has indeed found a significant main effect of the speed-alteration group, F(1, 14) = 4.95, p = .043, indicating that the mental-rotation improvement of the upper 3-qunatile subjects of the experiment group (M = 1137.6ms, SE = 182.8ms) was indeed significantly higher than the improvement of the control group’s upper 3-quantile subjects (M = 1712.90ms, SE = 182.8ms), which hints at a distinct mental-imagery improvement following virtual sensory-motor manipulation. However, despite the found significance, these results should be considered cautiously, as taking the 3-qunatile subset means narrowing the analysis to only 16 subjects out of the 48, thus significantly reducing its power.

## Discussion

In the current experiment we studied the relationship between individual mental-imagery and motor-planning capacities, in several motor-control modes. We assumed that mental imagery and motor planning share similar mechanism and that the extent of the overlap can be augmented by creating a novel sensorimotor context. We hypothesized that such novelty would induce a shift in the motor system towards the forward model, being more suitable in cases when the sensorimotor conditions need to be learned due to the lack of reliable predictions. We created a novel sensorimotor context by altering the speed of the artificial hand in our experimental settings. Our results showed that the chosen experimental settings created a set of conditions under which motor planning could be induced and measured, namely, that the time window between the stimulus appearance and the actual motion execution within the motor-control blocks indeed captured the motor planning processes, most notably in the open-loop motor-control block. The highest correlations between the average before-motion period durations (motor-control blocks) and the average mental-rotation RT’s (mental-rotation pretest and posttest blocks) were registered for the open-loop motor control blocks, supporting our fundamental finding that motor planning within the open-loop motor control setting is the most predictive of the mental imagery capacity. In addition, we found that under novel sensorimotor context, the mental-imagery-motor-planning capacity correlations were significantly higher than under the regular sensorimotor context, supporting our assumption that the shift towards the forward-model of the motor system would increase the overlap between mental imagery and motor planning processes.This increase was most pronounced in the open-loop motor control block, where the correlation increased from r = 0.47 under the regular context to r = 0.89 under sensorimotor alteration.

The cerebellar forward-model’s sensory-prediction is proposed as the underlying mutual mechanism involved in both generating mental imagery and enabling sensorimotor internal simulations in motor control and planning, predominantly when fast adjustment to novel sensorimotor environments is required. We suggest that the increased correlations between mental imagery, as reflected in the mental-rotation RTs, and motor planning durations in the open-loop blocks, under the altered hand speed condition, demonstrate an increased use of the forward-model for motor-planning, while adapting to the new sensorimotor context imposed by the hand speed manipulation. The lack of the hand speed alteration effect on the mental imagery speed, as reflected in the unchanged mental-motor correlations when comparing pretest and posttest measurements, indicates that a clean manipulation of the sensorimotor context was induced with the effect confined to the motor system, in line with previous reports^47,48^. The increase in the imagery-motor correlations therefore stems from consistent modifications in the motor control models attempting to adapt to the imposed change^27,49,50^. We propose, in line with the previous reports, that the adaptation to a novel sensorimotor context is gradually implemented^51^. Introducing novel environment makes the existing inverse-model inadequate to form a motor plan and a shift towards the forward-model occurs^52,53^. The forward-model is trained to predict the novel sensory outcomes of the motor commands, and only later, while using the already trained forward-model for motor control, the inverse-model is gradually adapting and forming a new sensory-goal-to-motor-command model which enables ongoing fluid and automatic motor operation in the new environment^32^. These dynamics would imply that the increase in the imagery-motor correlation should be followed by a decrease, when enough training is achieved for the inverse-model to learn the new sensory-motor rules to produce adequate motor outcome. The open-loop motor control is the optimal condition to demonstrate the shift from the inverse to the forward-model within the motion planning period, since in the absence of a relevant ongoing feedback, there is no need to correct the motion during its execution, so the motion is fully planned before the actual onset^54,55^. It is suggested that mental imagery relies on the internal forward model to generate predictions of future sensory states based on internal (efferent) simulation of motor commands, applied within the current sensory state, real or imagined. This ongoing sequence of simulated behavior within a simulated sensory context is conjectured to be the content of mental imagery^14,56–58^.We suggest, that under sensorimotor adaptation conditions, the motor-planning stage of an open-loop type of motion increasingly relies on the same forward-model mechanism that is used for mental imagery, and the individual patterns of the model’s activity (speed, accuracy) affect both similarly. This increased similarity is reflected in the increased correlation between individual motor-planning and mental-imagery capacities.

Despite the robustness of our key results, some methodological limitations of the experiment may question its sufficiency for our claimed interpretations. First, the existence of a virtual “embodiment” effect was assumed, without any of the commonly used measurements to validate it, which means different subjects may have experienced it to different degrees, if at all. Therefore, attributing the results to the general phenomena of embodiment, beyond the particular setup of this experiment, should be further validated in follow-up studies. Second , the order of the blocks was fixed rather than counterbalanced as commonly required. Keeping the same order of the blocks allowed the utilization of the unique nature of the motor interactions in each block, a proper experience of the sensorimotor environment and uninterrupted motor adaptation state, which might have been less effective with counterbalanced block order (e.g. when beginning with the mental-loop’s no-feedback mode). In addition, it was necessary to obtain the mental rotation measurements before (pretest) and after (posttest) the motor blocks, enforcing a fixed order of these blocks as well. The fixed block order is a potential confound in the interpretation of the results specifically related to the differences between the blocks. However, since the found effects interacted with the experimental groups even though all the participants received the same block order, this somewhat alleviates our concerns. Nonetheless, this is still a limitation that should be better addressed in future experiments.

The use of a single sensorimotor alteration is also somewhat limiting our interpretations, implying that the results might be manipulation-specific. Based on the relevant literature, we see no particular reason to assume that only this particular speed re-mapping, rather than other sensorimotor alterations, would lead to the found results. However, other types of sensorimotor modifications should be tested to examine the generalizability of our findings. In addition, the chosen experimental tasks, of mental and manual rotation, can potentially limit the association of the found effects with the overall capacities of mental-imagery and motor-planning. Mental rotation can be implemented through a number of systems, including motor imagery, visual imagery^59,60^, or by using an analytic non-rotation-based solution strategy^61^, and therefore may not be guaranteed to always represent and measure the general case of mental-imagery capacity. In addition, it can be claimed that since manual rotation and mental rotation share a similar task, the correlation between the motor planning and the mental-rotation capacity may directly stem from this similarity rather than representing a general case of individual motor/mental capacity. It can be further pointed out that the motor-planning stage in this experiment may include a sub-stage of mental-rotation, in order to first choose the best rotation direction even before planning the motion. In this case, high correlations between the motor-planning stage and the mental-rotation RTs are expected by design. However, the significant differences in the mental-motor correlations of the two experimental groups, with the control group correlations low-to-moderate at most, indicate that the increased correlations of the experimental group cannot be attributed to the task similarity alone. In addition, the main findings relate to the difference between the groups and the motor-control modes, rather than to the absolute correlations, further supporting our interpretations despite these experimental limitations.

Considering the potential of our findings, alongside the aforementioned experimental and methodological limitations, several follow-up studies are necessary. To address the constraints of the specificity and similarity of the mental and motor tasks, it would be suggested to incorporate a non-rotational motor task, and a more general mental-imagery task into the experimental design. In addition, to substantiate the link between the current results and the sensorimotor adaptation processes, a variety of sensorimotor alterations should be introduced, some dissociating the physical and virtual motion altogether (e.g. mapping physical non-rotational-motion to virtual rotation). To directly challenge the interpretation of the current results as related to an increased involvement of the forward-model in sensory prediction as part of the motor-planning when sensorimotor adjustment is required, one may manipulate the stability of the sensorimotor context. This can be achieved by comparing a condition where the sensorimotor alteration remains constant and predictable, thus enabling gradual adaptation to it, with another condition where the alteration is constantly changing, making the sensorimotor context unstable. Under this condition the forward-model’s fast learning would be disrupted, and the motor system would remain in its initial adaptation stage. These conditions might refine the contribution of the mental-motor shared predictive processes, as they are expected to be involved in the first but not in the second condition, whereas the rest of the factors potentially contributing to the increased mental-motor correlations, should not be affected by this manipulation. To test the applicability of a true seamless and natural motion-based cognitive assessment based on the found high correlations, one may run an experiment in VR where the motor task is simply to grab various objects as they appear in various angles. In a virtual sensorimotor altered environment (e.g. speed or angle re-mapping of the hand’s motion), we would expect that the time measured from object appearance to the motion onset towards it, will enable prediction of individual mental-imagery capacities. Moreover, as more participants will experience this VR task/game, the inter-subject predictive model will become increasingly better, which will potentially also lead to extracting novel theoretical insights from the emerging mental-motor patterns.

## Methods

### Participants

52 subjects, aged between 19-45 years (M = 28.2, SD = 6.9), colleagues or students, recruited through ads at the Technion campus, the internet and via two dedicated sessions in industry firms (Intel - n = 5; Elbit - n = 5), participated in the experiment. Subjects who performed the mental-rotation same/mirror task under 70% accuracy (2 subjects), or with mean RT> 9000ms (2 subjects), were excluded. All the subjects participated either voluntarily (10 subjects) or for payment of 30 NIS (42 subjects). There were 19 females and 41 right-handed participants. For each participant a lack of known attention-deficits or motor disorders was confirmed. All participants reported of having normal or corrected-to-normal vision. Participants were divided into two groups: 1. Altered hand speed - test group; 2. Regular hand speed - control group. Subjects were informed about the experimental setup and its use in the experiment, as well as on the entire experimental flow, but not about the two experimental groups and the purpose of the study. Informed consent was received from all participants, and the study was approved by the Technion Ethics Committee. The experiment was performed in accordance with relevant guidelines and regulations.

### Apparatus

The experimental setup consisted of a virtual-reality (VR) headset with an external head-tracking camera (Oculus-Rift DK2), headphones, and two hand-held motion-tracked controllers with a magnetic-field base-station (Razer Hydra). The VR experiment was developed using the C# programming language on the Unity3D game-engine and ran on a dedicated desktop-grade laptop (Asus ROG G750JHA, i7-4700HQ CPU, 16GB RAM, NVIDIA GeForce GTX 780m GPU). Participants sat in front of a table on which the VR camera and the controllers’ tracking base-station were positioned, with the hands comfortably resting either on the table or on the chair arms. Participants were instructed to place their hands so that a free motion of the hand-held controllers can be carried out. The virtual environment was built and programmed using a combination of self-designed and internet-purchased 3D models. It simulated a setting where an individual is sitting in front of a table in the middle of a 10x10 meters wooden deck outdoors, holding a controller-operated device. The setting was experienced as a 3D environment surrounding the subject. The head-tracking system induced a convincing sense of immersion and “presence”, by enabling looking around and moving the head and the seated body freely and realistically.

### Stimuli

**Figure 7:**
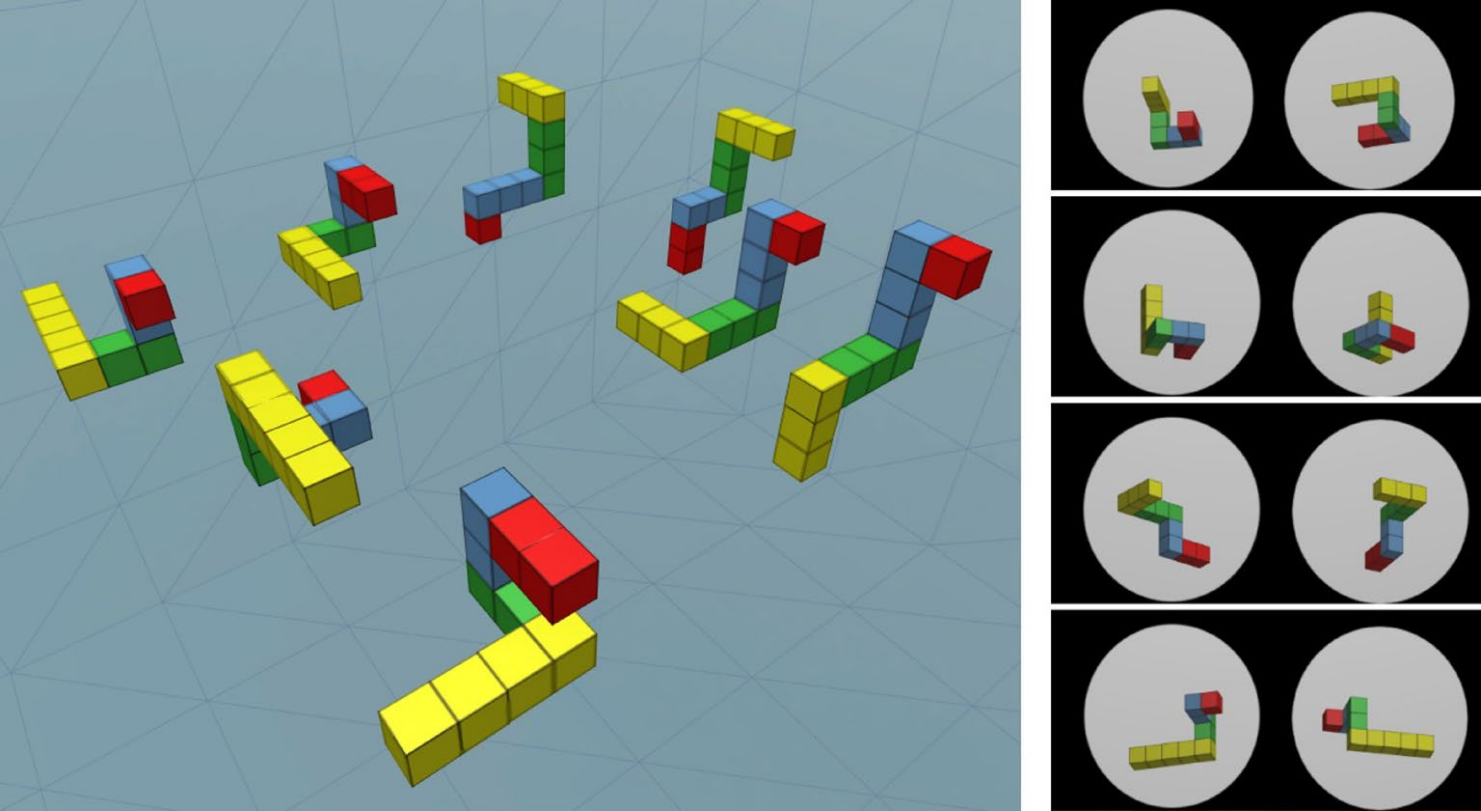
Mental-rotation block-shape stimuli.

The stimuli in the current study were pairs of images of three-dimensional (3D) block shapes, similar to the ones used in Shepard & Metzler’s mental-rotation experiment^62^. The block shapes were built in a 3D engine (Unity3D) and their respective images were generated as a 2D projection of the shapes when viewed from an angle below them to realistically match the angle of the virtual rotation device. Eight 3D four-arm cube shapes, in four possible rotations around their vertical main axis, 10/60/110/160 degrees, were used in the experiment (see Fig. 7). These were tested in a pilot experiment and chosen from a variety of options from a pre-validated set of shapes^63^, to maximize mental-rotation strategy and maintain balanced task-related difficulty and visual non-ambiguity. The absolute viewing angle of the shapes was also controlled to assure that all the shape arms are clearly visible and not co-occluding.

The cubes, comprising the shape arms, were colored uniformly and consistently across the shapes, to facilitate a quick comparison between the matching arms of the pairs of shapes presented in the mental/motor rotation task, and eliminate attempts to match the arms before the mental/motor rotation and the visual comparison are carried out. This addition encouraged the between-shape comparison and minimized the time spent on other processes, allowing the widely validated mental-rotation task, to be fully focused on testing the conjectured connections between mental-imagery and motor-control processes. Participants were informed, prior to the experiment, that the shapes were 3D rotated, and in some cases also mirrored, and that the rotation was applied only around the vertical axis. Introducing this information was intended to minimize the reported occasional use of cognitive strategies other than mental-rotation^61,64^.

### Procedure

The experiment consisted of five task blocks, with pretest and posttest mental-rotation blocks preceding and following, respectively, three motor-control blocks. The blocks were always in the same order, without any counterbalancing (explained further in the Discussion). Each block started with training trials, 20 in the first block and 10 in all the others, with a correct/incorrect feedback presented after each trial. The training trials were followed by a series of 40 test trials with no feedback presented. The transition from the training to the test part was clearly indicated by a large virtual sign, informing the participant that the training session has ended and that he/she may proceed to the next part when ready. After finishing each experimental block, subjects were instructed to put the controllers on the table and remove the headset and headphones for at least 1 minute of relaxation before proceeding to the next block.

In the mental rotation blocks, each trial began with the presentation of two randomly rotated block-shape images. The images in each pair could be either identical or mirrored. For each pair, subjects were asked to decide, as quickly as possible, whether they are identical or mirrored and press the right or left controller’s trigger button, respectively. The trial structure in each of the 3 motor-control blocks is illustrated in Fig. 3. In the motor-control blocks, participants had to quickly rotate the right shape via the right controller to match the left shape’s angle, under each block’s specific instructions. There were 3 types of motor-control blocks, open-loop, closed-loop and mental-loop, aimed to induce different motor control modes.

#### Open-loop motor-control block - “Rotate-to-Snap”

On each trial two images of a single 3D shape appear at different rotation angles. Participants are instructed to perform a rotation movement of the right shape to match its rotation angle with the left shape’s, as fast as possible. When the two shapes become identical in terms of their rotation angle, a click sound is heard, and the right shape’s rotation automatically stops (“snaps” into the matching angle), indicating the end of the current trial. Thus, in this block, participants learn that the goal is to quickly decide on the shortest rotation direction, clockwise or counterclockwise, and to rotate fast in the chosen direction without having to control for precision online, as the rotation snaps and stops automatically when the shapes’ rotation angles become identical. This presumably leads to an open-loop motor control, where the motor plan approximates the sufficient initial force to apply on the rotated hand, ahead of the motion, with no (or minimal) online visual or proprioceptive feedback utilized. Since there is no reliance on online feedback, we expected the motor planning stage in this block to be the longest among the blocks and to most significantly encode the information of the following actual motion.

#### Closed-loop motor-control block: “Rotate-to-Match”

In this block as well, two images of a single 3D shape appear at different rotation angles, and participants are asked to rotate the right shape to match its rotation angle with the left shape’s, as fast as possible. However, in this block the right shape does not snap and stop automatically when matching the left shape’s angle, but rather the subject has to manually adjust the right shape’s angle to precisely achieve the matching position in accordance with his/her judgement. Participants are asked to indicate that the matched position has been achieved by pressing the right controller’s trigger button and then proceed to the next trial. Consequently, in this block, participants have to both plan the direction and speed of the upcoming hand rotation as well as implement closed-loop control throughout the rotation, using visual and proprioceptive online feedback, until the shapes are matched (in accordance with their judgement) and the hand movement can be stopped. Since participants are aware that the planned motion will be further tuned based on online closed-loop control, we expected this mode to have shortened planning times, with the motor plan presumably encoding only part of the information of the following actual motion trajectory.

#### Mental-loop motor-control block: “Rotate Invisible”

In this block, on each trial the right shape is presented alone for 500 milliseconds, and only after the right shape disappears, the left shape is presented. Participants have to then rotate the “invisible” right shape, by executing a physical rotation of the hand, guided by the concurrent mental imagination of the right shape’s changing angle, until estimating that it reached the angle that matches the angle of the left shape, and then to press the right controller’s trigger button to confirm. After pressing the trigger, the right shape appears back again in its updated final angle, which gives the subject a visual feedback to evaluate his/her precision of the mentally-guided rotation. Then the two shapes disappear and the next trial begins. As a result, participants plan the hand’s rotation based on the visual image of the left shape and the dynamic mental image of the changing right shape. They continuously update the estimated right shape’s mental image and use closed-loop control, based on this image, in order to adjust the hand’s speed profile until mentally matching the two shapes angles. We have termed this unique motor-control mode, “mental-loop”, and we presumed it would uniquely induce an online coupling between the motor system during the execution of hand rotation and the concurrent mental-imagery updating of the angular state of the rotated invisible right shape. Since no actual visual feedback was available to the subject, we expected the motor plan to encode most of the following motion trajectory, while still relying on online mental closed-loop control during the actual motion.

### Statistical analyses

This experiment was designed to assess the mental-motor capacity correlations and their sensitivity to virtual embodiment with sensorimotor alteration. The data were analysed using a mixed-effects model, with two experimental groups defined in accordance with the implementation of the sensorimotor manipulation: altered hand speed - test group, regular hand speed - control group. For each participant the following data were obtained: (1) RTs from the two mental-rotation blocks (pretest and posttest); (2) RTs from the three motor-control blocks, separately for the different block types (open-loop, closed-loop, mental-loop); (3) Duration of each of the 3 segmented motion stages during motor-control trials (before motion, during motion, after motion), separately for each block type. The research hypotheses focused on the motor-planning stage, therefore, most of the analyzes were carried out on the before-motion stage duration without comparisons with the other stages.

The mental-rotation angular-disparity effect in the mental-rotation blocks was analyzed following Shepard & Metzler’s^62^. The shapes’ angular-disparity angle in each trial was used as a continuous measurement even though we used four specific angular disparities (10, 60, 110, 160 degrees). In the motor-control blocks the angular disparity was randomized from 30-90 degrees range in a continuous manner. The boundaries of the range were defined as 30 and 90 degrees, since manually rotating beyond 90 degrees is physically difficult, whereas rotation below 30 degrees is perceived as requiring almost no motion. We analyzed the mental and motor angular disparity to confirm our assumptions regarding the mental-rotation paradigm in VR and the motor-planning relation to actual motion. However, as we were primarily interested in the individual capacities, our main analyses were performed on the RTs averaged for each individual across the angular disparities.

### Motion kinematics

**Figure 8:**
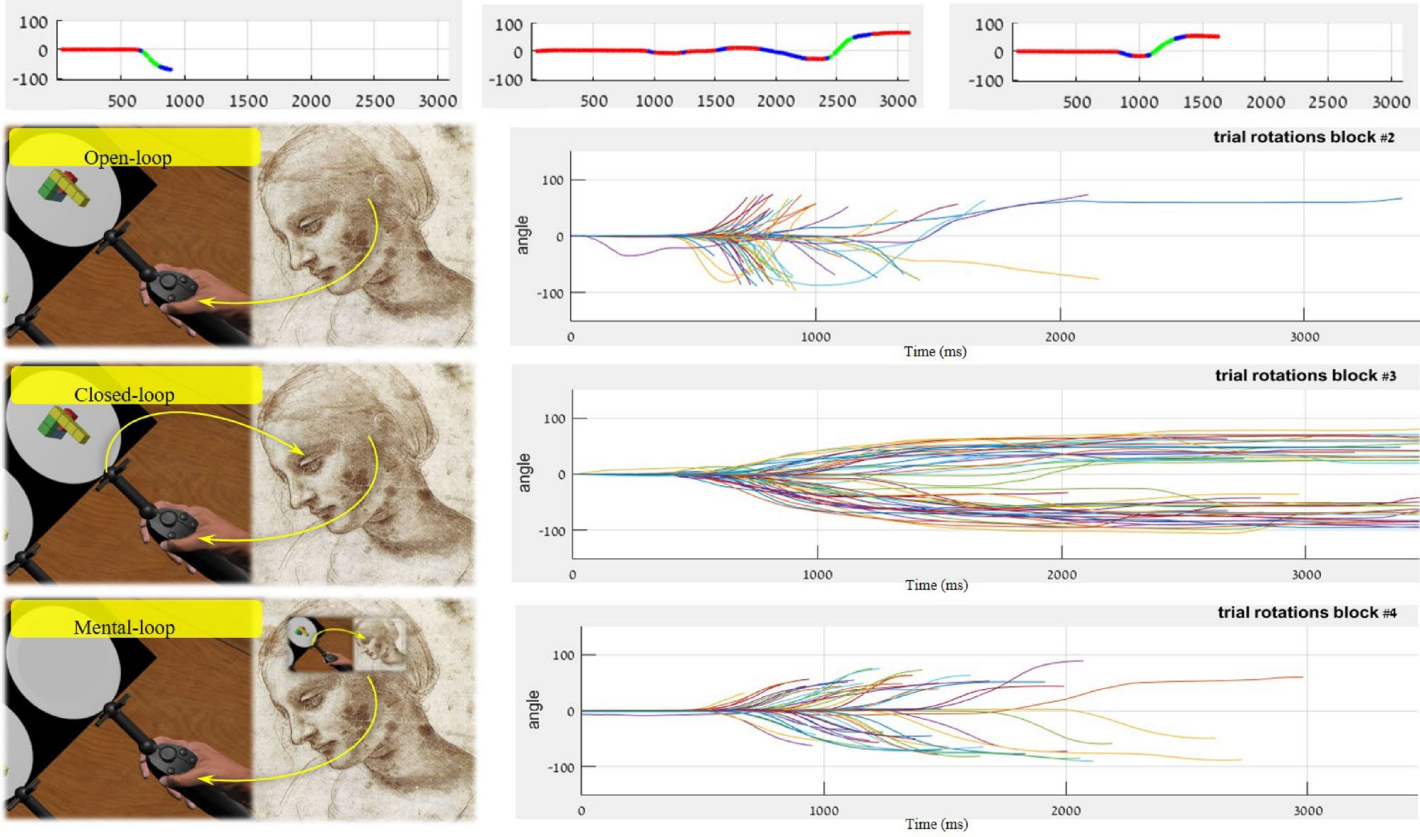
The motion kinematics of three representative trials and their segmentations (top) and all the trials of a single subject, shown per block. All the rotation movements, defined as the angle as a function of time, in each trial and each block, were automatically analyzed and segmented to extract the before-, during- and after-rotation durations. The segmentation of 3 trials is shown in the top plots, where green marks a rotation segment and red marks no-rotation (blue marks borderline parts). A full capture of all the trials of a single subject is shown in the 3 bottom plots. Time=0 marks the stimuli appearance. Note the differences in the motion stage durations and variability between the blocks.

Motion kinematics were obtained and analysed for each of the three motor-control blocks. A trial-by-trial analysis was performed to test the relation between the mental planning and the physical motion processes. Motion kinematics averaged for each individual across each block were analyzed to assess the motor and cognitive capacities. For each of the motor-control blocks, open-loop, closed-loop and mental-loop, each trial was segmented into the before-motion, during-motion and after-motion stages. For each stage, the kinematics were extracted, and duration means calculated. All these calculations were done using the MATLAB software (ver. R2018b). An example analysis for a single subject is shown in Fig. 8. The before-motion duration was the parameter of most interest in this research, as it is associated with the duration of action preparation and is assumed to reliably reflect the duration of motor-planning brain processes.

### Statistical Methods

Mental-rotation RTs were obtained only for the correct same-shape trials, to avoid measuring additional, mental-rotation unrelated, confounding factors, which may be included in the RTs of the mirror-shape trials or incorrect trials. We used either Analysis of variance (ANOVA), linear mixed-models, or correlation analysis, depending on the context and the tested question. For the linear mixed-models analysis, we treated the participants as a random factor nested within the hand speed manipulation groups, as well as their interactions with the shapes’ angular disparity factor. Consequently, our analysis calculated the percent of RT-variance accounted for by the individual mental-rotation capacity of the (randomly) chosen subjects, and only then tested for the statistical significance of the fixed-effects on the remaining unexplained RT variance. We used this approach to maximize the model’s explanatory power, while minimizing the interference of possibly confounding factors from the statistically tested effects. For the mixed-models analysis we used the Restricted Maximum Likelihood (REML) method, which is now the mainstream fitting method^65^, with a Kackar-Harville correction^66^ and a Kenward-Roger calculation of fixed-effects’ degrees-of-freedom denominator^67^. Where needed, T-tests and Tukey HSD were used for post-hoc pairwise comparisons, with correction of the alpha level to account for the respective family-wise error rate. Baseline significance threshold of p< 0.05 was used in all analyses. All the statistical analyses were carried out using JMP, Statistical analysis$^{\mathrm{TM}}$13.0, from SAT, data analysis software.
